# Occurrence and Washout of Health‐Hazardous Chemicals in Children's Clothing

**DOI:** 10.1111/cod.70170

**Published:** 2026-04-25

**Authors:** Awat Dostberg, Tim Åström, Ioannis Sadiktsis, Conny Östman, Ulrika Nilsson

**Affiliations:** ^1^ Department of Chemistry Stockholm University Stockholm Sweden

**Keywords:** ATD‐GC/MS, children's clothes, screening, sweat migration, textile chemicals, washout

## Abstract

**Background:**

Textile chemicals may constitute a hazardous exposure and lead to skin sensitization or other health problems. Children, due to their thinner, less developed skin, are more susceptible to this exposure.

**Objectives:**

To investigate the occurrence and levels of 50 textile chemicals in children´s skin‐close clothing. Further, to investigate the washout effect of these textile chemicals and their tendencies to migrate from the textile fibres into artificial sweat.

**Methods:**

Screening of 60 children's clothes purchased on the Swedish retail market was performed using coupled automated thermal desorption‐gas chromatography/mass spectrometry (ATD‐GC/MS).

**Results:**

Among the most frequently occurring chemicals were non‐regulated quinolines, halogenated arylamines, phthalates, and nitrobenzenes. The highest concentrations were found for benzyl benzoate, 1400 μg/g, and 2‐bromo‐4,6‐dinitroaniline, 300 μg/g. The highest number and levels of chemicals were detected in garments made of 100% polyester, while the fewest and lowest levels were determined in light‐coloured cotton. Laundry experiments revealed that cotton garments had the greatest washout effect, whereas most of the chemical content remained in 100% polyester garments even after 10 laundry cycles.

**Conclusions:**

Results indicate a lower exposure from the investigated cotton garments, especially after laundry. On the other hand, cotton exhibited threefold greater chemical migration into artificial sweat than polyester. The strong dependence on fibre material is important to consider when estimating the bioaccessible chemical exposure from garments and related health risks.

## Graphical abstract

A survey of 60 children's garments from the Swedish market showed a varying, often highly complex chemical content. The highest levels were generally found in synthetic materials and the lowest in light‐coloured cotton. Further, the washout effect by laundry and sweat migration of textile chemicals was investigated.

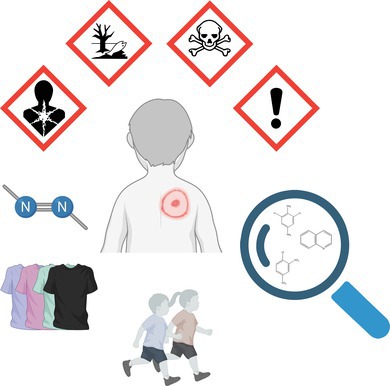

## 1 Introduction

The complex mixtures of chemicals commonly found in clothing may constitute health risks. Many of these substances are non‐covalently attached to the fibres [1, 2, 3, 4, 5, 6, 7, 8] and can migrate and be absorbed through human skin, a process facilitated by rubbing and sweating [9, 10, 11, 12, 13]. Depending on concentration and uptake, this exposure may lead to skin sensitisation and/or different systemic health effects. Disperse dyes are considered some of the main skin sensitisers in textiles, but textiles may contain a wide variety of other proven or suspected allergens [14, 15, 16, 17, 18, 19, 20, 21, 22, 23].

The complexity of the chemical content in textiles results from the use of process chemicals in many manufacturing steps, such as bleaching, dyeing, softening, and the addition of flame‐ or water‐resistant chemicals, as well as the unintentional introduction of contaminants and byproducts. Arylamines, unreacted precursors from azo dye synthesis, are examples of unintentional chemicals with no function in finished synthetic garments [24]. It is desirable to remove as many of these substances as possible, for example, by laundering. However, in a previous study, quinolines and benzothiazole were found to be only partially removed from newly purchased garments, even after several laundry cycles [25].

Regulatory frameworks in the European Union currently only restrict a limited number of harmful textile chemicals, such as 22 arylamines and their associated azo dyes, due to their documented carcinogenicity [26]. The limit set for these individual arylamines is 30 μg/g, and 50 μg/g for quinoline [27]. In addition, a few potentially endocrine‐disrupting phthalates, including dibutyl phthalate, benzyl butyl phthalate, and diisobutyl phthalate, are limited to a total concentration of 1000 μg/g [28]. Many potentially hazardous chemicals remain unregulated, thus constituting a potential gap in consumer safety [25].

Apart from arylamines, several unregulated halogenated nitrobenzenes have recently been shown to occur as contaminants in common synthetic clothing [22]. Two of these substances, 2,4‐ and 2,5‐dinitrochlorobenzene, are strong sensitisers, and 3,5‐dinitrobromobenzene has been shown to exhibit mutagenicity in the Ames test [23].

The aim of the present study was to investigate children's clothing from the Swedish retail market for the occurrence of 50 textile chemicals, including quinolines, phthalates, halogenated arylamines, nitrobenzenes, and benzothiazole. Analyses were performed using automated thermal desorption–gas chromatography/mass spectrometry (ATD‐GC/MS) [29]. The washout effect of laundering was investigated for 10 selected garments, as well as the bioaccessibility of textile chemicals in terms of migration from the fibres into artificial sweat.

## 2 Methods

### 2.1 Chemicals

Acetonitrile (HPLC grade) was purchased from Honeywell International Inc. (Charlotte, NC, USA). Deactivated (silanised) glass wool of pesticide grade was obtained from Supelco (Bellefonte, PA). Internal standards (IS) and reference compounds that were used are listed in the Supporting Information (Table S1). All reference compounds were of higher purity than 95% according to GC/MS analyses. The cold trap in the ATD was packed with Tenax‐TA (PerkinElmer Inc., Waltham, MA, USA).

### 2.2 Standard Solutions

Stock solutions of standard and surrogate internal standard (IS) compounds were prepared in acetonitrile and stored at −24°C under an argon atmosphere to prevent oxidation or degradation of the compounds. A mixed standard solution with concentrations around 16 μg/mL of the individual analytes was prepared in acetonitrile. The IS solutions were also diluted to around 16 μg/mL in acetonitrile prior to use.

### 2.3 Garments

Sixty garments were purchased from six retail stores at open markets in the Stockholm area in 2022. Details for all garments are found in the Supporting Information (Table S2). Except for buttons, zippers and four garments which had prints, each individual textile was uniform in colour, material and pattern.

### 2.4 Sample Preparation for ATD‐GC/MS Analysis

Pieces from three randomly selected parts of the investigated garment were cut into smaller pieces (approx. 0.25 cm^2^) and pooled. Printed parts were excluded from sampling. Approximately 10–30 mg of the sample was weighed and added to an ATD desorption tube, following the previously described procedure [29]. Internal standard mixture (10 μL, 16 μg/mL) was added to the sample inside the tube and allowed to dry at room temperature for 2 h before ATD‐GC/MS analysis [29].

### 2.5 Laundry

Ten garments were selected for investigating the washout effect of chemicals, as detailed in Table 1. They were selected from the 60 garments based on the largest number and the highest amount of chemicals present. The laundry program was set to 40°C for 60 min, finalising with centrifugation at 1200 rpm. Around 25 g of fragrance‐free detergent (Via Classic Colour Sensitive, Unilever Sverige AB, Stockholm, Sweden) was used per laundry cycle. After washing, the garments were dried outside the machine prior to analysis, following the sample preparation and procedure as described in Section 2.4. Each selected garment was washed separately once, five times, and 10 times.

**TABLE 1:** Details of the 10 garments selected for the laundry experiment.

Garment no.	Composition	Country of production	Colour
3	93% Polyester—7% Elastane	China	Red
5	100% Recycled polyester	Bangladesh	Black
6	82% Recycled polyester—18% Elastane	China	Blue
7	95% Polyester—5% Elastane	China	Pink
9	71% Cotton—26% Polyester—3% Elastane	Bangladesh	Green
11	91% Polyester—19% Elastane	Bangladesh	Orange
27	100% Cotton	Bangladesh	Green
28	95% Polyester—5% Elastane	Cambodia	Violet
32	100% Polyester	China	Dark blue
45	93% Polyester—7% Elastane	China	Orange

### 2.6 Instrumental Analysis

The ATD‐GC/MS method for textile analysis has been described previously [29, 30]. Briefly, each sample was thermally desorbed at 175°C for 10 min, with the nitrogen desorption flow set to 50 mL/min and the cold trap inlet split set to 49 mL/min. Both the inlet valve and the transfer line were set to 270°C, and the cold trap was held at −10°C. The secondary desorption, that is, the desorption of the cold trap, was performed at 260°C for 5 min with a He flow of 11.3 mL/min and an outlet split of 10 mL/min. As a result, approximately 0.25% of the chemical vapour desorbed from the textile was transferred to the GC column. The GC oven program was initially held at 90°C for 0.5 min, followed by a 10°C/min ramp to 130°C, held for 2 min. A second ramp of 10°C/min was used to reach a final temperature of 325°C, which was held for 12 min. The total analysis run time was 38 min. The MS was run in EI mode at 70 eV in full scan mode from 40 to 550 m/z, with a scan time of 200 ms and an inter‐scan delay of 50 ms. The filament was off during the first 4 min of the GC program. MS transfer line temperature was set to 325°C, and the ion source temperature was 200°C.

### 2.7 Test of Contamination From the Laundry Machine

Around 1 g of the rubber seal inside the laundry machine was cut and extracted with 15 mL of acetone using ultrasonication at 40°C for 60 min. The extract was evaporated and reduced under nitrogen at 40°C to a final volume of 3 mL. Fifty microlitres of extract was added to approximately 25 mg of conditioned blank polyester, inserted into an ATD desorption tube. A volume of 50 μL of quinoline‐d_7_ was added and the samples left to dry at room temperature for 2 h. Each sample was analysed in triplicate with the procedure described in Section 2.6.

### 2.8 Test of Chemical Migration From Textiles to Artificial Sweat

Approximately 1 g of a blank textile sample was cut into 1 × 1 cm^2^ pieces, put into a round‐bottom flask, and 200 μL of a standard mixture was added to the sample, which was left to dry at room temperature for 2 h. The standard mixture contained 24 compounds, including quinolines, phthalates, anilines and nitrobenzene at concentrations around 160 μg/mL. Then, 100 mL of artificial sweat containing sebum was added to each sample, and the samples were kept at 35°C for 16 h. After clean‐up, the sweat extract was analysed with GC/MS. The procedure was performed in triplicate for both a cotton and a polyester blank textile, that is, garments with no previously detected textile chemicals. The preparation of artificial sweat and cleanup were performed as described previously [23, 31, 32, 33]. Details of the 24 substances, results, and analysis are provided in the Supporting Information (Tables S3 and S4).

### 2.9 Quantification

The amount of each analyte in each sample was determined using the absolute response factors of closely eluting IS compounds [29]. Further, to control the ATD‐GC/MS instrumental performance, n‐eicosane‐d_42_ was added to each sample, as this compound has been shown to be fully desorbed from all types of garment composition at 175°C [29]. In addition, analyses were performed on QC samples composed of 100% pure polyester and spiked with 40 ng of each analyte, IS, and n‐eicosane‐d_42_. The QC samples were, prior to spiking, pre‐conditioned at 200°C for 20 min to remove any detectable contaminants [29].

## 3 Results and Discussion

### 3.1 Screening of Children's Clothing

A screening of 50 chemicals in 60 selected garments was conducted in this survey. The clothes were obtained directly from the retailers and not washed before the screening. The used ATD‐GC/MS method is applicable for a wide range of semi‐volatile textile chemicals with lipophilicities (logP) and molecular weights that enable skin uptake [9, 34]. Another advantage is the solvent‐free, simplified sample preparation, which requires very little manual handling, benefiting repeatability and time efficiency. A relative standard deviation of less than 10% is typically obtained for most investigated compounds. When including all compounds hitherto investigated, the coefficient of variation (CV) is below 20%. As with all other analytical methods, ATD‐GC/MS also has limitations. It cannot be used for low‐volatility compounds or thermolabile chemicals and is applicable neither to metals nor to most disperse dyes. Disperse azo dyes have been shown to be degraded to arylamines at desorption temperatures exceeding 175°C [30].

The wide variety of selected textile compositions and colours, representing most of the apparel on the Swedish retail market, is a strength of the present study. To the best of our knowledge, similar surveys of children's clothing monitoring such a broad range of potentially skin‐sensitising chemicals have not been conducted to date. A limitation is that all garments in the present survey were purchased within a relatively short period during 2022, thus representing only a snapshot of chemical profiles, while new materials and chemicals may have been introduced to the market since then. A larger sample size, including additional fibre types and producers, would likely lead to a better representation of the whole market. Still, the present study is important, as it highlights the need for further surveys and control of potential skin sensitisers in textiles, particularly in children's clothing.

Table 2 shows the frequencies and concentration ranges found for the 30 substances detected in the 60 garments. The method detection limits with the ATD‐GC/MS method at S/*N* = 3 were 0.005–0.02 μg/g for quinolines, 0.01–0.4 μg/g for arylamines, 0.02–0.14 μg/g for nitrobenzenes, and 0.01–0.30 μg/g for phthalates [29].

**TABLE 2:** Frequency of occurrence and concentration ranges (μg/g) of quantified chemicals in 60 children's clothes (*n* = 3, CV < 20%).

Compounds	Detection frequency %	Concentration range (μg/g)	Colour of the garment containing highest level	Material composition
Benzothiazole	83	0.063–4.8	Orange	91% Polyester—19% elastine
Diisobutyl phthalate	65	0.10–3.2	Blue—brown	100% Cotton
Quinoline	47	0.037–75	Black	100% Recycled polyester
Benzyl benzoate	42	0.072–1400	Dark blue	83% Cotton—17% polyester
Dimethyl phthalate	35	0.024–0.20	Red—pink	100% Recycled polyester
Dimethyl terephthalate	30	0.043–4.4	Red—pink	100% Recycled polyester
2,6‐Dichloro‐4‐nitroaniline	28	0.47–35	Black	100% Recycled polyester
Isoquinoline	27	0.049–27	Black	100% Recycled polyester
2‐Chloro‐4‐nitroaniline	27	0.15–23	Red	93% Polyester—7% elastane
4‐Methylquinoline	25	0.019–3.7	Black	100% Recycled polyester
2‐Chloro‐4,6‐dinitroaniline	23	1.2–250	Black	100% Recycled polyester
8‐Methylquinoline	20	0.016–6.5	Black	100% Recycled polyester
3‐Methylquinoline	20	0.029–5.2	Black	100% Recycled polyester
2‐Methylquinoline	15	0.10–14	Black	100% Recycled polyester
6‐Methylquinoline	18	0.034–15	Black	100% Recycled polyester
1‐Chloro‐3,5‐dinitrobenzene	18	0.46–92	Black	100% Recycled polyester
2,6‐Dimethylquinoline	13	0.022–2.5	Black	100% Recycled polyester
2,4‐Dimethylquinoline	13	0.23–2.5	Black	100% Recycled polyester
2‐Bromo‐4,6‐dinitroaniline	13	1.1–300	Dark blue	83% Cotton—17% polyester
4‐Nitroaniline	10	0.31–8.9	Violet—Brown—white	100% Polyester
2,4‐Dinitroaniline	10	0.48–12	Black	100% Recycled polyester
1‐Bromo‐3,5‐dinitrobenzene	10	0.10–16	Dark blue	83% Cotton—17% polyester
4‐Chloro‐2‐nitroaniline	7	0.76–14	Red	100% Cotton
2,6‐Dibromo‐4‐nitroaniline	7	0.67–54	Dark blue	100% Recycled polyester
3,4‐Dichloroaniline	5	0.036–3.9	Violet—Brown—white	100% Polyester
Diphenylamine	2 garments	0.049 and 0.076	Black—white	95% Recycled polyester—%5 elastine
1‐Chloro‐2,4‐dinitrobenzene	2 garments	1.7 and 22	Black	100% Recycled polyester
Dipropyl phthalate	1 garment	0.087	Violet—Brown—white	100% Polyester
Benzyl butyl phthalate	1 garment	3.5	Pink	80% Recycled polyester—20% elastine
2,6‐Dichlorobenzene‐1,4‐diamine	1 garment	0.16	Red	93% Polyester—7% elastine

*Note:* The detailed results are shown in the Table S5. The method's quantification limit was set to S/*N* = 5.

**FIGURE 1:**
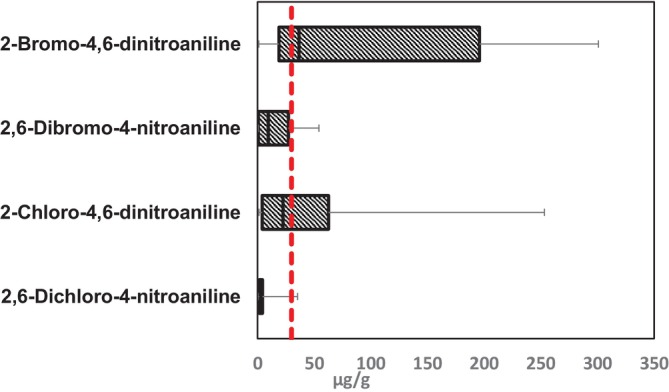
Currently unregulated arylamines found at the highest levels in the present survey. The red line shows the current REACH limit of 30 μg/g for 22 other similar, regulated arylamines.

**FIGURE 2:**
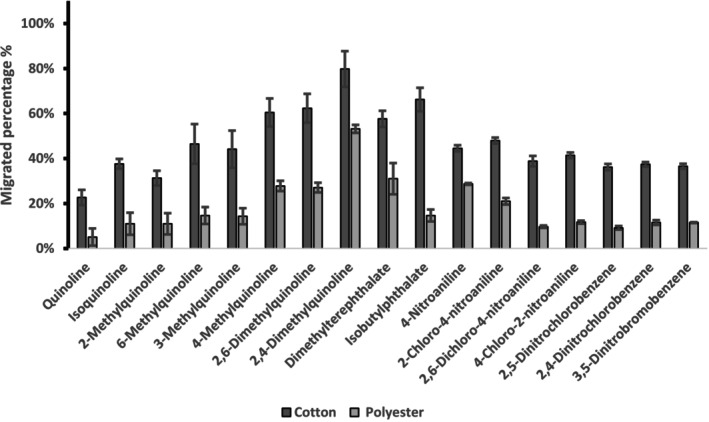
Migrated percentage of individual substances from spiked blank polyester and blank cotton garments to artificial sweat. Results for all 24 spiked substances are found in the Supporting Information (Table S3).

Arylamines and halogenated nitrobenzenes were among the most frequently detected compounds, several of which have reported mutagenic properties [1]. However, none of the 22 arylamines hitherto regulated within the EU could be detected. As illustrated in Figure 1 and 2‐bromo‐4,6‐dinitroaniline far exceeded the EU level for other regulated arylamines (30 μg/g) in several garments. In one garment, a pair of dark blue pants made of 83% cotton and 17% polyester, the amount of 2‐bromo‐4,6‐dinitroaniline was 300 μg/g, and in another garment, 2‐chloro‐4,6‐dinitroaniline exceeded the 30 μg/g limit by 8‐fold. The source of arylamines is likely impure azo dyes used in the manufacturing process [22]. Halogenated nitrobenzenes have previously been reported as textile chemicals in a survey of Swedish clothing [23]. In the present study, one of these compounds, 1‐bromo‐3,5‐dinitrobenzene, which has recently been shown to be mutagenic in the Ames test [23], was identified in six of the garments (10%), all of dark colours. Four of these were made of 100% polyester, and two contained a high percentage of cotton. Another isomer, 1‐chloro‐2,4‐dinitrobenzene, since long known as a strong skin sensitiser, was detected in a black jacket made of 100% recycled polyester and manufactured in Bangladesh.

The skin‐sensitisation effect is currently unknown for most of the identified chemicals. ECHA reports harmonised CLP classifications of Skin Sens 1 for 3,4‐dichloroaniline (EC 202–448‐4), 1‐chloro‐2,4‐dinitrobenzene (EC 202–551‐4), and 1‐bromo‐3,5‐dinitrobenzene (EC 804–805‐5). In Table 3, these data are included together with predicted skin sensitisation obtained using in silico software (Derek Nexus v.6.3.0, Lhasa Ltd., Leeds, UK), an expert‐rule decision‐tree model based on structural alerts. Derek reports predicted sensitisation in the following order of reasoning: Probable > Plausible > Equivocal. One of the compounds reported as a skin sensitiser by ECHA, 1‐bromo‐3,5‐dinitrobenzene, was predicted as equivocal by Derek. This compound was previously found to be non‐reactive in the peptide reactivity assay (DPRA) [23]. In the same study, 2,6‐dichlorobenzene‐1,4‐diamine was found to be a moderate skin sensitiser, based on both DPRA and the human cell line activation test (h‐CLAT), a result which agrees better with the Derek prediction. The most abundant arylamines (Figure 1) were all predicted to be equivocal sensitisers, while no harmonised skin sensitisation classification under CLP applies to these substances. Altogether, this shows that further studies are needed to clarify their roles as potential textile allergens.

**TABLE 3:** In silico prediction on skin sensitisation and ECHA data, respectively, for the 30 chemicals quantified in this study.

Substances	Hazard category for skin sensitisers (annex VI to the CLP regulation)^a^	Derek prediction
Benzothiazole	—	Non‐sensitiser
Quinoline	—	Non‐sensitiser
Isoquinoline	—	Non‐sensitiser
2‐Methylquinoline	—	Non‐sensitiser
8‐Methylquinoline	—	Non‐sensitiser
6‐Methylquinoline	—	Non‐sensitiser
3‐Methylquinoline	—	Non‐sensitiser
4‐Methylquinoline	—	Non‐sensitiser
2,6‐Dimethylquinoline	—	Non‐sensitiser
2,4‐Dimethylquinoline	—	Non‐sensitiser
4‐Chloro‐2‐nitroaniline	—	Equivocal
4‐Nitroaniline	—	Equivocal
2‐Chloro‐4‐nitroaniline	—	Equivocal
2,6‐Dichloro‐4‐nitroaniline	—	Equivocal
2‐Chloro‐4,6‐dinitroaniline	—	Equivocal
2,6‐Dibromo‐4‐nitroaniline	—	Equivocal
2,4‐Dinitroaniline	—	Equivocal
2‐Bromo‐4,6‐dinitroaniline	—	Equivocal
3,4‐Dichloroaniline	Skin Sens 1	Equivocal
Diphenylamine	—	Non‐sensitiser
Dimethyl phthalate	—	Non‐sensitiser
Dimethyl terephthalate	—	Non‐sensitiser
Dipropyl phthalate	—	Non‐sensitiser
Benzyl benzoate	—	Plausible
Diisobutyl phthalate	—	Non‐sensitiser
Benzyl butyl phthalate	—	Equivocal
1‐Chloro‐3,5‐dinitrobenzene	—	Equivocal
1‐Chloro‐2,4‐dinitrobenzene	Skin Sens 1	Probable
2,6‐Dichlorobenzene‐1,4‐diamine	—	Plausible
1‐Bromo‐3,5‐dinitrobenzene	Skin Sens 1	Equivocal

^a^ According to the Global Harmonised System (GHS) and the Classification, Labelling, and Packaging (CLP) regulation, skin sensitisers for which potency data are missing and thus cannot be subcategorised as 1A/1B should be classified as Skin Sens 1 [35].

Benzothiazole, predicted to be a non‐sensitiser, was most frequently detected and measured in 50 garments (83%). This compound is a large‐volume industrial chemical used in many applications. The exact source is difficult to determine, but since this chemical is used for vulcanisation of rubber, it could originate from various machines with seals or other rubber components. Benzothiazole is considered an emerging contaminant in the environment and in organisms, but limited information is available on its health effects in humans [36]. Phthalates were also frequently detected (up to 65% detection frequency), as well as quinolines (up to 47% detection frequency). Phthalates, as is benzothiazole, are predicted as non‐sensitisers. As they are used as plasticisers, they may originate from different stages of textile manufacturing, such as printing [37]. Lab utensils and solvents may also contaminate the analyses with phthalates, which makes blank sample analyses crucial. Quinolines, also predicted to be non‐sensitisers, are ubiquitous in textiles, according to several previously published surveys [2]. This compound class originates from petroleum and may therefore already be included in polyester synthesis. Quinolines may also be used as precursors in dye synthesis and as solvents in dyeing processes [38]. The highest level of quinoline in the present study was 75 μg/g determined in a black shirt made of 100% recycled polyester (Table 2), a level well above the EU limit of 50 μg/g. The level is of concern since this compound is a human carcinogen [27]. Several other quinolines, such as the non‐regulated isoquinoline and methyl quinolines with possibly similar toxicities, could also be quantified; however, at individual levels below the regulated limit. The health effects of these chemicals might be similar, but none has so far been reported/investigated. The highest individual concentration was found for benzyl benzoate. This chemical is widely used in the perfume, pharmaceutical, and food industries. It is reported as an irritating substance by ECHA [39, 40, 41].

The most complex mixtures of targeted chemicals, including 15 to 19 analytes, were found in five garments, all made of 100% polyester, three of which were recycled polyester. All these textiles were dark in colour. For most synthetic garments, the number of quantified chemicals ranged from 5 to 14. The lowest numbers, 1–2 substances, as well as generally the lowest concentrations, were detected in white cotton garments.

### 3.2 Wash‐Out of Chemicals

An intuitive measure to lower the risk of skin exposure to harmful textile chemicals is to wash newly bought clothes. To estimate the washout effect of the chemicals detected in this study, 10 garments (Table 1) were selected based on both complexity and the highest measured levels. The garments were washed separately for 1, 5 and 10 cycles. As shown in Table 4, most of the chemicals in the synthetic garments fully remained even after 10 laundry cycles. Some levels increased for several chemicals, but the reasons for these increases were not further investigated. A possible explanation for the large increase in halogenated arylamines is the chemical reduction of azo dyes facilitated by enzymes or chemicals in the detergent. The exceptional increase of both dimethyl terephthalate and benzothiazole could be due to contaminants from the laundry machine, water tubing, or detergent. A piece of the rubber seal from the washing machine was extracted and analysed (Section 2.7). The results indicate this could be one of the main sources, with a measured benzothiazole content of 1.3 μg/g.

**TABLE 4:** The highest concentration measured of each chemical among the 10 selected newly bought 100% synthetic clothes, the garment ID number and the level (%) remaining after 1, 5 and 10 laundry cycles (*n* = 3).

Compound	Non‐washed (μg/g)	Garment ID	1 cycle (%)	5 cycles (%)	10 cycles (%)
Quinoline	10.5 ± 1.2	5	99.7 ± 13.7	118 ± 33	94.6 ± 19.5
Isoquinoline	27.8 ± 0.58	5	106 ± 6	109 ± 2	112 ± 1
2‐Methylquinoline	8.61 ± 0.27	5	111 ± 3	113 ± 2	114 ± 1
2,4‐Dimethylquinolines	1.83 ± 0.05	5	106 ± 5	107 ± 1	110 ± 2
2‐Chloro‐4,6‐dinitroaniline	258 ± 18	5	107 ± 16	123 ± 6	127 ± 2
2,6‐Dichloro‐4‐nitroaniline	24.7 ± 3.2	32	122 ± 6	212 ± 14	170 ± 8
2‐Chloro‐4‐nitroaniline	54.7 ± 4.9	32	112 ± 7	149 ± 9	137 ± 10
2‐Bromo‐4,6‐dinitroaniline	5.59 ± 0.93	5	97 ± 9	114 ± 16	117 ± 8
2,6‐Dibromo‐4‐nitroaniline	4.15 ± 0.58	5	123 ± 8	132 ± 3	135 ± 2
Dimethyl phthalate	0.640 ± 0.07	6	22.0 ± 4.5	18.4 ± 0.1	15.5 ± 0.8
Dimethyl terephthalate	0.947 ± 0.13	32	110 ± 25	331 ± 12	201 ± 36
Diisobutyl phthalate	6.80 ± 1.5	6	67.0 ± 0.8	39.8 ± 0.7	19.2 ± 0.7
Benzyl benzoate	11.1 ± 2.2	5	7.17 ± 1.04	7.90 ± 0.80	6.95 ± 0.71
4‐Nitroaniline	5.16 ± 0.5	5	106 ± 7	111 ± 4	118 ± 4
1‐Chloro‐2,4‐dinitrobenzene	9.43 ± 0.8	5	128 ± 13	130 ± 8	131 ± 3
Benzothiazole	3.43 ± 0.14	11	298 ± 9	3360 ± 189	2000 ± 300

*Note:* The garment IDs are shown in Table S1, and the details of the wash‐out investigation results for individual synthetic garments are shown in the Tables S6–S13.

A garment of 71% cotton and 29% synthetic fibres (garment ID 9), containing lower levels of chemicals, showed higher overall chemical removal efficiency (Table 5), except for quinolines. The reason for the differences between quinolines and other chemicals was not further examined. However, one reason could be that the quinolines are mostly attached to the polyester fibres in this garment and therefore are difficult to remove, similar to 100% synthetic garments. A similar behaviour was previously observed for quinolines in polyester garments [25] The increase of diisobutyl phthalate is most likely due to contamination.

**TABLE 5:** Concentrations of chemicals detected in a garment of 71% cotton and 29% synthetic fibres (garment 9) and the relative levels (%) remaining after 1, 5 and 10 laundry cycles (*n* = 3).

Compound	Non‐washed (μg/g)	1 cycle (%)	5 cycles (%)	10 cycles (%)
Quinoline	1.23 ± 0.13	121 ± 2	112 ± 6	102 ± 16
Isoquinoline	0.483 ± 0.05	114 ± 9	89.8 ± 6.2	90.1 ± 10.1
2‐Methylquinoline	0.166 ± 0.01	102 ± 5	99.1 ± 6.2	102 ± 22
6‐Methylquinoline	0.120 ± 0.002	104 ± 11	91.9 ± 3.4	95 ± 10
2,4‐Dimethylquinoline	0.186 ± 0.02	118 ± 9	110 ± 12	103 ± 0
Dimethyl phthalate	0.267 ± 0.001	44.3 ± 8.5	14.0 ± 2.0	15.5 ± 5.9
Dimethyl terephthalate	0.314 ± 0.03	102 ± 1	55.0 ± 5.4	63.8 ± 4.5
4‐Chloro‐2‐nitroaniline	1.65 ± 0.17	12.9 ± 2.5	15.8 ± 0.3	12.2 ± 5.1
4‐Nitroaniline	0.554 ± 0.11	3.64 ± 1.7	4.03 ± 1.98	2.79 ± 1.31
Benzyl benzoate	1.87 ± 0.08	44.3 ± 0.7	61.7 ± 3.3	32.7 ± 0.6
Diisobutyl phthalate	0.505 ± 0.03	166 ± 3	98.2 ± 5.2	52.4 ± 2.8
Benzothiazole	1.06 ± 0.05	117 ± 7	260 ± 4	246 ± 4

The investigated 100%‐cotton garment contained the fewest chemicals. It also exhibited the highest washout effect for quinolines (Table 6). The greater washout effect observed for arylamines and quinolines from garments with higher cotton content may be explained by the disruption of hydrogen bonds between nitrogen atoms and cellulose hydroxyl groups under aqueous conditions. For benzyl benzoate, on the other hand, the washout release was slow.

**TABLE 6:** Concentrations of chemicals detected in a garment made of 100% cotton (garment ID 27) and the relative levels (%) remaining after 1, 5 and 10 laundry cycles (*n* = 3).

Compound	Non‐washed (μg/g)	1 cycle (%)	5 cycles (%)	10 cycles (%)
Quinoline	1.64 ± 0.3	16.3 ± 4.9	16.2 ± 6.0	7.5 ± 1.5
Isoquinoline	0.414 ± 0.3	ND	ND	ND
Dimethyl phthalate	0.258 ± 0.06	ND	ND	ND
Dimethyl terephthalate	0.132 ± 0.02	ND	ND	ND
Benzyl benzoate	0.705 ± 0.04	113 ± 60	101 ± 15	80.9 ± 19.2
Benzothiazole	0.427 ± 0.09	34.1 ± 1.6	72.2 ± 18.4	205 ± 40

Abbreviation: ND, not detected.

### 3.3 Migration of Chemicals From Garments to Artificial Sweat

The results from the laundry experiments indicate that the extent to which the studied chemicals are released into sweat and become bioaccessible also depends on the type of fabric. In a report from the General Federal Institute for Risk Assessment (BfR) from 2012 [42], the worst‐case migration into sweat from hydrophilic and hydrophobic textiles, respectively, is estimated to 2% for the former and 0.1% for the latter. However, a recent investigation of polyester, a hydrophobic fibre material, revealed that some native halogenated textile chemicals migrated into artificial sweat to a much higher extent, up to 39% [23]. In the present study, we therefore tested the migration using spiked chemicals on blank cotton and polyester since the levels of native compounds were generally very low in the cotton garments in the present survey. This was considered reasonable, as we have previously shown that the investigated analytes adsorb onto the textile fibres similarly to their corresponding native chemicals [29].

Figure 2 shows the results from sweat migration of spiked textile chemicals from 100% cotton and 100% polyester, respectively. The migration from cotton is consistently higher than from polyester, in most cases by a factor ≥ 3. The migration of 2,4‐dimethylquinoline was found as high as 80%. For polyester, the halogenated chemicals 2‐chloronitroaniline (around 20%) and 2,6‐dichloro‐4‐nitroaniline (around 10%) showed results close to those previously reported for the corresponding native chemicals [23], verifying the validity of using spiked chemicals in the present study. However, the present experiment concerns only two different types of fibres, while other materials may behave differently. The present study stresses the importance of investigating the migration factors for exposure and risk assessments.

## 4 Conclusions

In this survey, dark‐coloured garments of polyester and recycled polyester showed the highest concentrations and numbers of chemicals, while white cotton garments contained the fewest and lowest amounts of the targeted chemicals. None of the 22 arylamines regulated by REACH was detected, in compliance with the regulations. On the other hand, several other arylamines of similar structures, but less studied and of unknown health effects, were shown to occur frequently and often above the set levels for regulated arylamines. A high detection frequency was observed for quinolines and nitrobenzenes. Although the skin‐sensitisation effects remain unknown for most of the identified compounds, this is of particular concern given the high levels and large numbers of these chemicals in children's clothing. It shows that human exposure to hazardous chemicals can begin at a very young age. Several of the studied chemicals have previously been shown to penetrate human skin, and uptake may be higher in young children due to their thinner, less developed skin. The role of these chemicals in textile allergy should thus be further studied.

Laundry showed a very limited removal of arylamines and quinolines from synthetic fibres. The high levels and number of chemicals emphasise the need for expanded control and regulation. In this survey, the best choices among the investigated garments to limit the exposure of children seem to be those made of light‐coloured 100% cotton since this material both contained much less of the chemicals as well as had a much higher washout effect.

Sweat migration experiments revealed large differences in migration rate between polyester and cotton. The migration factors should be investigated in exposure and risk assessments. Even if the cotton garments investigated in the present survey were shown to contain less of chemicals, thus implying lower possible exposure, the higher migration factors of cotton, on the other hand, may increase the risk.

## Author Contributions

**Ulrika Nilsson:** funding acquisition, project administration, writing – review and editing, resources, supervision. **Awat Dostberg:** conceptualization, formal analysis, methodology, investigation, validation, visualization, writing – original draft, data curation. **Tim Åström:** formal analysis, investigation, methodology, writing – review and editing. **Conny Östman:** writing – review and editing, supervision. **Ioannis Sadiktsis:** writing – review and editing, supervision.

## Funding

This work was supported by the Stockholms Universitet, the Stiftelsen för Miljöstrategisk Forskning (Mistra SafeChem Grant No 2018/11), the Svenska Forskningsrådet Formas (2021‐01540) and the HORIZON EUROPE European Research Council (BioSusTex 101135372).

## Conflicts of Interest

The authors declare no conflicts of interest.

## Supporting information


**Table S1:** Reference compounds used in this study with the purities given by the suppliers. † = > 95% purity according to GC/MS analysis.
**Table S2:** Details of the 60 garments used in this survey.
**Table S3:** The result of the migrated amounts of 24 substances from spiked cotton and spiked polyester to artificial sweat, analysed with GC/MS, and clean up performed using SPE (Oasis MCX, 6 cc/500 mg, Waters, Milford, MA, USA). The recoveries of the studied substances ranged from 56% to 129%. The mixture of internal standards compensated for losses, and all CVs were below 12%.
**Table S4:** The retention times and target ions of the internal standards used in analysing the migrated amount of chemical from cotton and polyester to artificial sweat using GC/MS.
**Table S5:** Results of the target screening of 60 garments have been analysed with ATD‐GC/MS.
**Table S6:** The quantified amount of target substances and the ratio of the amount after one, five, and ten laundry cycles in garment.
**Table S7:** The quantified amount of target substances and the ratio of the amount after one, five, and ten laundry cycles in garment.
**Table S8:** The quantified amount of target substances and the ratio of the amount after one, five, and ten laundry cycles in garment.
**Table S9:** The quantified amount of target substances and the ratio of the amount after one, five, and ten laundry cycles in garment.
**Table S10:** The quantified amount of target substances and the ratio of the amount after one, five, and ten laundry cycles in garment.
**Table S11:** The quantified amount of target substances and the ratio of the amount after one, five, and ten laundry cycles in garment.
**Table S12:** The quantified amount of target substances and the ratio of the amount after one, five, and ten laundry cycles in garment.
**Table S13:** The quantified amount of target substances and the ratio of the amount after one, five, and ten laundry cycles in garment.

## Data Availability

The data that supports the findings of this study are available in the Supporting Information of this article.
