# Supplementary material for: Occurrence and Washout of Health‐Hazardous Chemicals in Children's Clothing
Source: Contact Dermatitis. 2026 Apr 25;95(2):148–58. doi: 10.1111/cod.70170 (PMC13327236; doi:10.1111/cod.70170)
Supplement: Supplementary file 1 — Table S1: Reference compounds used in this study with the purities given by the suppliers. † = > 95% purity according to GC/MS analysis. Table S2: Details of the 60 garments used in this survey. Table S3: The result of the migrated amounts of 24 substances from spiked cotton and spiked polyester to artificial sweat, analysed with GC/MS, and clean up performed using SPE (Oasis MCX, 6 cc/500 mg, Waters, Milford, MA, USA). The recoveries of the studied substances ranged from 56% to 129%. The mixture of internal standards compensated for losses, and all CVs were below 12%. Table S4: The retention times and target ions of the internal standards used in analysing the migrated amount of chemical from cotton and polyester to artificial sweat using GC/MS. Table S5: Results of the target screening of 60 garments have been analysed with ATD‐GC/MS. Table S6: The quantified amount of target substances and the ratio of the amount after one, five, and ten laundry cycles in garment. Table S7: The quantified amount of target substances and the ratio of the amount after one, five, and ten laundry cycles in garment. Table S8: The quantified amount of target substances and the ratio of the amount after one, five, and ten laundry cycles in garment. Table S9: The quantified amount of target substances and the ratio of the amount after one, five, and ten laundry cycles in garment. Table S10: The quantified amount of target substances and the ratio of the amount after one, five, and ten laundry cycles in garment. Table S11: The quantified amount of target substances and the ratio of the amount after one, five, and ten laundry cycles in garment. Table S12: The quantified amount of target substances and the ratio of the amount after one, five, and ten laundry cycles in garment. Table S13: The quantified amount of target substances and the ratio of the amount after one, five, and ten laundry cycles in garment. [file COD-95-148-s001.docx]

**Supplementary Material**

**Occurrence and washout of health-hazardous chemicals in children´s clothing**

Awat Dostberg, Tim Åström , Ioannis Sadiktsis, Conny Östman, Ulrika Nilsson*

Department of Chemistry, Stockholm University, SE-106 91 Stockholm, Sweden

* Corresponding author:
Email: unils@su.se

Phone: +46-(0)70-224-0851

**TABLE OF CONTENTS**

**Table S1** Reference compounds used in this study

**Table S2**: Details of the 60 garments used in this study

**Table S3**: The result of the migrated amounts of 24 substances from spiked cotton and spiked polyester to artificial sweat, analysed with GC/MS

**Table S4**: The retention times and target ions of the internal standards used in analysing the migrated amount of chemical from cotton and polyester to artificial sweat using GC/MS

**Table S5**: Results of the target screening of 60 garments have been analysed with ATD-GC/MS

**Table S6**: The quantified amount of target substances and the ratio of the amount after one, five, and ten laundry cycles in garment 5

**Table S7**: The quantified amount of target substances and the ratio of the amount after one, five, and ten laundry cycles in garment3

**Table S8**: The quantified amount of target substances and the ratio of the amount after one, five, and ten laundry cycles in garment 6

**Table S9**: The quantified amount of target substances and the ratio of the amount after one, five, and ten laundry cycles in garment 7

**Table S10**: The quantified amount of target substances and the ratio of the amount after one, five, and ten laundry cycles in garment 11

**Table S11**: The quantified amount of target substances and the ratio of the amount after one, five, and ten laundry cycles in garment 28

**Table S12**: The quantified amount of target substances and the ratio of the amount after one, five, and ten laundry cycles in garment 32

**Table S13**: The quantified amount of target substances and the ratio of the amount after one, five, and ten laundry cycles in garment 45

**Table S1:** Reference compounds used in this study with the purities given by the suppliers. † = > 95% purity according to GC/MS analysis.

| Compound | CAS | Molecular formula | Molecular mass (Da) | | Purity | Supplier |
| --- | --- | --- | --- | --- | --- | --- |
| Quinoline-d_7_ | 34071-94-8 | C9D7N | 136 | 98% | | Cambridge Isotopes, Tewksbury, USA |
| 2-Methylbenzothiazole | 120-75-2 | C8H7NS | 149 | 99% | | Sigma Aldrich, St Louis, USA |
| 3-Nitroaniline-d_4_ | 115044-52-5 | C6D4H2N2O2 | 142 | 98% | | Cambridge Isotopes, Tewksbury, USA |
| Diethyl phthalate-d_4_ | 93952-12-6 | C12D4H10O4 | 226 | 98% | | Cambridge Isotopes, Tewksbury, USA |
| 4-Nitroaniline-15N_2_ | 119516-81-3 | C6H6N2O2 | 140 | 98% | | Sigma Aldrich, St Louis, USA |
| Benzophenone-d10 | 22583-75-1 | C13D10O | 192 | 99% | | Sigma Aldrich, St Louis, USA |
| 2,4-Dinitrobromobenzene-d₃ | 1313734-81-4 | C6H3BrN2O4 | 250 | 98% | | Sigma Aldrich, St Louis, USA |
| Bis(2-ethylhexyl) phthalate-d4 | 93951-87-2 | C24D4H34O4 | 394 | 98% | | Cambridge Isotopes, Tewksbury, USA |
| 4,4-Dihydroxybiphenyl-d**8** | [612480-60-1](https://www.medchemexpress.com/cas/612480-60-1.html) | C12H2D8O2 | 194 | 99% | | CDN Isotopes, Quebec, Canada |
| n-Eicosane-d42 | 62369-67-9 | C20D42 | 324 | 98% | | Sigma Aldrich, St Louis, USA |
| Quinoline | 91-22-5 | C9H7N | 129 | 97% | | Merck, Darmstadt, Germany |
| Isoquinoline | 119-65-3 | C9H7N | 129 | 97% | | Sigma Aldrich, St Louis, USA |
| 2-Methylquinoline | 91-63-4 | C10H9N | 143 | 95% | | Sigma Aldrich, St Louis, USA |
| 8-Methylquinoline | 611-32-5 | C10H9N | 143 | 97% | | Sigma Aldrich, St Louis, USA |
| 6-Methylquinoline | 91-62-3 | C10H9N | 143 | N.S.^†^ | | Labkemi AB, Stockholm, Sweden |
| 3-Methylquinoline | 612-58-8 | C10H9N | 143 | 99% | | Sigma Aldrich, St Louis, USA |
| 4-Methylquinoline | 491-35-0 | C10H9N | 143 | 99% | | Sigma Aldrich, St Louis, USA |
| 2,6-Dimethylquinoline | 877-43-0 | C11H11N | 157 | N.S.^†^ | | K&K Laboratories, Cleveland, Ohio, USA |
| 2,4-Dimethylquinoline | 1198-37-4 | C11H11N | 157 | N.S.^†^ | | K&K Laboratories, Cleveland, Ohio, USA |
| 4-Chloroaniline | 106-47-8 | C6H6ClN | 127 | 98% | | Sigma Aldrich, St Louis, USA |
| 2-Bromoaniline | 615-36-1 | C6H6BrN | 171 | 98% | | Sigma Aldrich, St Louis, USA |
| 3-Bromoaniline | 591-19-5 | C6H6BrN | 171 | 98% | | Sigma Aldrich, St Louis, USA |
| 4-Bromoaniline | 106-40-1 | C6H6BrN | 171 | 97% | | Sigma Aldrich, St Louis, USA |
| 2-Nitroaniline | 88-74-4 | C6H6N2O2 | 138 | 98% | | Sigma Aldrich, St Louis, USA |
| 3,4-Dichloroaniline | 95-76-1 | C6H5Cl2N | 161 | 98% | | Sigma Aldrich, St Louis, USA |
| 3-Nitroaniline | 99-09-2 | C6H6N2O2 | 138 | 98% | | Sigma Aldrich, St Louis, USA |
| 2,6-dichloro-1,4-phenylenediamine | 609-20-1 | C6H6Cl2N2 | 176 | 90% | | Apollo Scientific, Stockport, UK |
| 4-Chloro-2-nitroaniline | 89-63-4 | C6H5ClN2O2 | 172 | 99% | | Sigma Aldrich, St Louis, USA |
| 4-Nitroaniline | 100-01-6 | C6H6N2O2 | 138 | 99% | | Sigma Aldrich, St Louis, USA |
| 2-Chloro-4-nitroaniline | 121-87-9 | C6H5ClN2O2 | 172 | 99% | | Sigma Aldrich, St Louis, USA |
| 2,6-Dichloro-4-nitroaniline | 99-30-9 | C6H4Cl2N2O2 | 206 | 96% | | Sigma Aldrich, St Louis, USA |
| Dibenzylamine | 103-49-1 | C14H15N | 197 | 97% | | Sigma Aldrich, St Louis, USA |
| 3,5-Dibromo-1,2-phenylenediamine | 1575-38-8 | C6H6Br2N2 | 266 | 95% | | Enamine Ltd, Kyiv, Ukraine |
| 2-Chloro-4,6-dinitroaniline | 3531-19-9 | C6H4ClN3O4 | 217 | 97% | | Sigma Aldrich, St Louis, USA |
| 2,6-Dibromo-4-nitroaniline | 827-94-1 | C6H4Br2N2O2 | 266 | 97% | | Sigma Aldrich, St Louis, USA |
| 2,4-Dinitroaniline | 97-02-9 | C6H5N3O4 | 183 | 98% | | Sigma Aldrich, St Louis, USA |
| 2-Bromo-4,6-dinitroaniline | 1817-73-8 | C6H4BrN3O4 | 263 | 94% | | Sigma Aldrich, St Louis, USA |
| 3,5-Dichloronitrobenzene | 618-62-2 | C6H3Cl2NO2 | 192 | 95% | | Sigma Aldrich, St Louis, USA |
| 1,4-Dinitrobenzene | 100-25-4 | C6H4N2O4 | 168 | 98% | | Sigma Aldrich, St Louis, USA |
| 1,3-Dinitrobenzene | 99-65-0 | C6H4N2O4 | 168 | 98% | | Sigma Aldrich, St Louis, USA |
| 1,2-Dinitrobenzene | 528-29-0 | C6H4N2O4 | 168 | 97% | | Sigma Aldrich, St Louis, USA |
| 2,4-Dinitrochlorobenzene | 97-00-7 | C6H3ClN2O4 | 202 | 99% | | Sigma Aldrich, St Louis, USA |
| 2,5-Dinitrochlorobenzene | 619-16-9 | C6H3ClN2O4 | 202 | N.S.^†^ | | Toronto Research Chemicals, Toronto, CA |
| 3,5-Dinitrobromobenzene | 18242-39-2 | C6H3BrN2O4 | 247 | N.S.^†^ | | Apollo Scientific, Stockport, UK |
| Dimethyl phthalate | 131-11-3 | C10H10O4 | 194 | 99% | | Sigma Aldrich, St Louis, USA |
| Dimethyl terephthalate | 120-61-6 | C10H10O4 | 194 | 99% | | Sigma Aldrich, St Louis, USA |
| Dipropyl phthalate | 131-16-8 | C14H18O4 | 250 | 98% | | Sigma Aldrich, St Louis, USA |
| Diisobutyl phthalate | 84-69-5 | C16H22O4 | 278 | 99% | | Sigma Aldrich, St Louis, USA |
| Benzyl butyl phthalate | 85-68-7 | C19H20O4 | 312 | 98% | | Sigma Aldrich, St Louis, USA |
| Bis(2-ethylhexyl) phthalate | 117-81-7 | C24H38O4 | 390 | 99% | | Sigma Aldrich, St Louis, USA |
| Bis(2-ethylhexyl) terephthalate | 6422-86-2 | C24H38O4 | 390 | 97% | | Sigma Aldrich, St Louis, USA |
| Naphthalene | 91-20-3 | C10H8 | 128 | 99% | | Honeywell, Charlotte, USA |
| 2-Phenoxyethanol | 122-99-6 | C8H10O2 | 138 | 99% | | Sigma Aldrich, St Louis, USA |
| Benzothiazole | 95-16-9 | C7H5NS | 135 | 96% | | Sigma Aldrich, St Louis, USA |
| Biphenyl | 92-52-4 | C12H10 | 154 | 99% | | Sigma Aldrich, St Louis, USA |
| 3-Nitrophenol | 554-84-7 | C6H5NO3 | 139 | 99% | | Sigma Aldrich, St Louis, USA |
| 4-Nitrophenol | 100-02-7 | C6H5NO3 | 139 | 99% | | Sigma Aldrich, St Louis, USA |
| 2,4-Dinitrophenol | 51-28-5 | C6H4N2O5 | 184 | 98% | | Sigma Aldrich, St Louis, USA |
| Benzophenone | 119-61-9 | C13H10O | 182 | 99% | | Sigma Aldrich, St Louis, USA |
| Benzyl benzoate | 120-51-4 | C14H12O2 | 212 | 99% | | Sigma Aldrich, St Louis, USA |
|  |  |  |  |  | |  |

**Table S2**: Details of the 60 garments used in this survey

| Garment | Color | Composition | Country of production | Type of clothes |
| --- | --- | --- | --- | --- |
| 1 | Red | 95% Cotton - 5% Elastane | Bangladesh | Body |
| 2 | Black- yellow | 84% Polyester - 16% Elastane | Bangladesh | Swimming short |
| 3 | Red | 93% Polyester - 7% Elastane | China | Dress |
| 4 | Pink | 92% Polyester - 8% Elastane | China | Dress |
| 5 | Black | 100% Recycled polyester | Bangladesh | Shirt |
| 6 | Blue - Gray | 82% Recycled polyester - 18% Elastane | China | Swimming short |
| 7 | Pink | 95% Polyester - 5% Elastane | China | Dress |
| 8 | Brown | 95% Recycled polyester - 5% Elastane | China | Leggings |
| 9 | Green | 71% Cotton - 26% Polyester - 3% Elastane | Bangladesh | Pant |
| 10 | White-Red- Green | 95% Cotton - 5% Elastane | Bangladesh | Body |
| 11 | Orange | 91% Polyester - 19% Elastane | Bangladesh | Swimming body |
| 12 | Black - Yellow | 100% Cotton | Bangladesh | Nightwear |
| 13 | Red | 100% Cotton | Bangladesh | Blus |
| 14 | Red | 95% Polyester - 5% Elastane | Cambodia | Dress |
| 15 | Black | 100% Recycled polyester | China | Nightwear |
| 16 | Blue - brown | 100% Cotton | Bangladesh | Nightwear |
| 17 | Red- Pink | 100% Recycled polyester | China | Blus |
| 18 | Black | 95% Organic cotton - 5% Elastane | Bangladesh | Body |
| 19 | Brown | 100% Cotton | Turkey | Blus |
| 20 | Yellow - black | 95% Cotton - 5% Elastane | Bangladesh | Short |
| 21 | Black | 82% Cotton - 14% Polyamide - 4% Elastane | Bangladesh | Leggings |
| 22 | White - Red | 100% Recycled polyester | India | Dress |
| 23 | Black - Yellow | 100% Cotton | India | Nightwear |
| 24 | Orange - white | 100% Cotton | Cambodia | Leggings and body |
| 25 | Black - white | 95% Recycled Polyester - 5% Elastane | China | Jacket |
| 26 | Darkgreen | 100% Polyester | China | Dress |
| 27 | Green | 100% Cotton | Bangladesh | Jacket |
| 28 | Violet | 95% Polyester - 5% Elastane | Cambodia | Leggings |
| 29 | Violet - Brown - White | 100% Polyester | China | Jacket |
| 30 | Dark blue | 84% Polyester - 16% Elastane | Bangladesh | T-shirt |
| 31 | Dark blue | 100% Recycled polyester | China | Gloves |
| 32 | Dark blue | 100% Polyester | China | Scarf |
| 33 | White - Pink | 100% Polyester | China | Dress |
| 34 | Violet | 95% Polyester - 5% Elastane | Cambodia | Blus |
| 35 | Black | 100% Wool | Bangladesh | Body |
| 36 | Red | %75Cotton - 25% Polyester | Bangladesh | Dress |
| 37 | Pink | 80% Recycled Polyester - 20% Elastane | China | Swimming body |
| 38 | Dark blue - Red-Green | 100% Viscose | India | Dress |
| 39 | Brown | 100% Polyester | China | Nightwear |
| 40 | Black - white | 95% Polyester - 5% elastane | China | Pant |
| 41 | Red | 94% Polyester - 6% elastane | India | Dress |
| 42 | Dark blue - white | %95 organic cotton - 5% elastine | China | Body |
| 43 | Red | 100% polyester | China | Dress |
| 44 | Dark blue | %83 Cotton - 17% polyester | Bangladesh | Pant |
| 45 | Orange | 93% Polyester - 7% elastane | China | Dress |
| 46 | Gray | 100% Organic cotton | Bangladesh | Body |
| 47 | Black | 92% Polyester - 8% Elastane | China | Pant |
| 48 | Blue | %95 Organic cotton - 5% Elastine | Bangladesh | Body |
| 49 | Darkblue | %80 Cotton - 20% Polyester | Bangladesh | Blus |
| 50 | Pink | 100% Cotton | Bangladesh | T-shirt |
| 51 | White | %95 Cotton - 5% Elastine | India | T-shirt |
| 52 | White | 100% Cotton | Bangladesh | T-shirt |
| 53 | White | 100% Cotton | Bangladesh | T-shirt |
| 54 | White | %95 Cotton - 5% Elastine | Bangladesh | Body |
| 55 | White | %75 Cotton - 23% Poly amide - 2% Elastine | Turkey | Socks |
| 56 | White | 100% Cotton | India | T-shirt |
| 57 | White | %80 Cotton - 20% Polyester | Bangladesh | Jacket |
| 58 | White | 66% Acrylic - 17% Poly amide - 10% polyester - 4% Wool - 3% Elastine | Myanmar | Jacket |
| 59 | White | %40 Polyester - 38% cotton - 16% polyamide - 6% Acrylic | Myanmar | Blus |
| 60 | White | 100% Polyester | China | Jacket |

**Table S3**: The result of the migrated amounts of 24 substances from spiked cotton and spiked polyester to artificial sweat, analysed with GC/MS, and clean up performed using SPE (Oasis MCX, 6cc/500 mg, Waters, Milford, MA, USA). The recoveries of the studied substances ranged from 56% to 129%. The mixture of internal standards compensated for losses, and all CVs were below 12%.

| Analytes | Amount Spiked (µg) | Volume spiked (µl) | Rt (min) | Target Ion | Migrated from cotton | STD | Migrated from polyester | STD |
| --- | --- | --- | --- | --- | --- | --- | --- | --- |
| Benzothiazole | 32 | 200 | 6.05 | 135 | 2% | 1% | < 1 % | - |
| Quinoline | 32 | 200 | 6.12 | 129 | 23% | 3% | 5% | 4% |
| Isoquinoline | 32 | 200 | 6.43 | 129 | 38% | 2% | 11% | 5% |
| 2-Methylquinoline | 32 | 200 | 6.87 | 143 | 31% | 3% | 11% | 5% |
| 8-Methylquinoline | 32 | 200 | 6.95 | 143 | 8% | 2% | 1% | 1% |
| 6-Methylquinoline | 32 | 200 | 7.52 | 143 | 46% | 9% | 15% | 4% |
| 3-Methylquinoline | 32 | 200 | 7.62 | 143 | 44% | 8% | 14% | 4% |
| 4-Methylquinoline | 32 | 200 | 7.96 | 143 | 60% | 6% | 28% | 2% |
| 2,6-Dimethylquinoline | 32 | 200 | 8.24 | 157 | 62% | 6% | 27% | 2% |
| 2,4-Dimethylquinoline | 32 | 200 | 8.63 | 157 | 80% | 8% | 53% | 2% |
| Dimethylphthalate | 32 | 200 | 8.90 | 163 | 46% | 5% | 50% | 5% |
| Dimethylterephthalate | 32 | 200 | 9.17 | 163 | 58% | 4% | 31% | 7% |
| Benzylbutyl-phthalate | 32 | 200 | 18.20 | 149 | 84% | 22% | 8% | 1% |
| Isobutylphthalate | 32 | 200 | 12.96 | 149 | 66% | 5% | 15% | 3% |
| Benzylbenzoate | 32 | 200 | 12.50 | 105 | 40% | 6% | 5% | 0% |
| 4-Nitroaniline | 32 | 200 | 11.65 | 138 | 45% | 1% | 29% | 0% |
| 2-Chloro-4-nitroaniline | 32 | 200 | 12.50 | 172 | 48% | 1% | 21% | 1% |
| 2,6-Dichloro-4-nitroaniline | 32 | 200 | 12.54 | 206 | 39% | 2% | 10% | 1% |
| 4-Chloro-2-nitroaniline | 32 | 200 | 11.19 | 172 | 41% | 1% | - | 1% |
| 2-Bromo-4,6-dinitroaniline | 32 | 200 | 16.20 | 261 | 22% | 12% | 13% | 1% |
| 3,4-Dichloroaniline | 32 | 200 | 9.00 | 161 | 14% | 1% | 4% | 1% |
| 2,5-Dinitrochlorobenzene | 32 | 200 | 10.71 | 202 | 36% | 1% | 9% | 1% |
| 2,4-Dinitrochlorobenzene | 32 | 200 | 10.92 | 202 | 37% | 1% | 12% | 1% |
| 3,5-Dinitrobromobenzene | 32 | 200 | 11.61 | 246 | 37% | 1% | 12% | 0% |

**Table S4**: The retention times and target ions of the internal standards used in analysing the migrated amount of chemical from cotton and polyester to artificial sweat using GC/MS

| Analytes | Rt (min) | Target Ion (m/z) |
| --- | --- | --- |
| Quinoline-d_7_ | 6.08 | 136 |
| 2-Methylbenzthiazole | 6.75 | 149 |
| 3-Nitroaniline-d_4_ | 9.88 | 142 |
| Diethylphthalate-d_4_ | 10.34 | 153 |
| Benzophenone-d_10_ | 11.04 | 110 |
| 4-Nitroaniline-N_15_ | 11.65 | 140 |
| 2,4-Dinitrobromobenzene-d₃ | 12.13 | 249 |
| 4,4-Dihydroxybiphenyl-d**8** | 16.11 | 194 |
| Bis(2-ethylhexyl) phthalate-d4 | 18.64 | 153 |

**Table S5**: Results of the target screening of 60 garments have been analysed with ATD-GC/MS

| Substances | Number | Frequency % | Range (µg/g) | Q1 (µg/g) | Q3 (µg/g) | Median (µg/g) |
| --- | --- | --- | --- | --- | --- | --- |
| Benzothiazole | 50 | 83 | 0.063 – 4.8 | 0.14 | 0.4 | 0.2 |
| Quinoline | 28 | 47 | 0.037 - 75 | 0.16 | 1.9 | 0.28 |
| Isoquinoline | 16 | 27 | 0.049 - 27 | 0.085 | 2.9 | 0.7 |
| 2-Methylquinoline | 9 | 15 | 0.1 – 14 | 0.13 | 1.8 | 0.9 |
| 8-Methylquinoline | 12 | 20 | 0.015 - 6.5 | 0.1 | 0.77 | 0.18 |
| 6-Methylquinoline | 11 | 18 | 0.034 - 15 | 0.3 | 1.7 | 0.8 |
| 3-Methylquinoline | 12 | 20 | 0.029 - 5.2 | 0.1 | 0.6 | 0.3 |
| 4-Methylquinoline | 15 | 25 | 0.019 - 3.7 | 0.09 | 0.79 | 0.2 |
| 2,6-Dimethylquinoline | 8 | 13 | 0.022 - 2.5 | 0.145 | 1.1 | 0.5 |
| 2,4-Dimethylquinoline | 8 | 13 | 0.23 - 2.5 | 0.31 | 2.3 | 0.8 |
| 4-Chloro-2-nitroaniline | 4 | 7 | 0.76 - 14 | 0.8 | 4.5 | 1.1 |
| 4-Nitroaniline | 6 | 10 | 0.31 - 8.9 | 0.883 | 5.7 | 4 |
| 2-Chloro-4-nitroaniline | 16 | 27 | 0.15 - 23 | 1.6 | 6.2 | 2.9 |
| 2,6-Dichloro-4-nitroaniline | 17 | 28 | 0.47 - 35 | 1.5 | 4.3 | 2.7 |
| 2-Chloro-4,6-dinitroaniline | 14 | 23 | 1.2 - 253 | 4 | 63 | 23 |
| 2,6-Dibromo-4-nitroaniline | 4 | 7 | 0.67- 54 | 0.7 | 27 | 9.5 |
| 2,4-Dinitroaniline | 6 | 10 | 0.48 - 12 | 1.7 | 8.9 | 2.6 |
| 2-Bromo-4,6-dinitroaniline | 8 | 13 | 1.1 - 300 | 19 | 195 | 37 |
| 3,4-Dichloroaniline | 3 | 5 | 0.036 - 3.9 | 0.13 | 2.1 | 0.2 |
| Diphenylamine | 2 | 2 garments | 0.049 and 0.076 | - | - | - |
| Dimethyl phthalate | 21 | 35 | 0.024 - 0.02 | 0.045 | 0.09 | 0.07 |
| Dimethyl terephthalate | 18 | 30 | 0.042 – 4.4 | 0.16 | 1.3 | 0.3 |
| Dipropyl-phthalate | 1 | 1 garment | 0.087 | - | - | - |
| Benzyl benzoate | 25 | 42 | 0.072 - 1408 | 0.16 | 21 | 0.6 |
| Diisobutyl phthalate | 39 | 65 | 0.1 - 3.2 | 0.35 | 1.2 | 0.6 |
| Benzyl butyl phthalate | 1 | 1 garment | 3.5 | - | - | - |
| 1-Chloro-3,5dinitrobenzene | 11 | 18 | 0.469 - 92.5 | 0.7 | 33 | 1.9 |
| 1-Chloro-2,4-dinitrobenzene | 2 | 2 garments | 1.7 and 22 | - | - | - |
| 2,6-Dichloro-benzenediamine | 1 | 1 garment | 0.16 | - | - | - |
| 1-Bromo-3,5-dinitrobenzene | 6 | 10 | 0.1 - 15 | 2.5 | 5 | 4.1 |

**Table S6**: The quantified amount of target substances and the ratio of the amount after one, five, and ten laundry cycles in garment 5

| Compounds | Amount (µg/g) | 1 Cycle (%) | 5 Cycles (%) | 10 Cycles (%) |
| --- | --- | --- | --- | --- |
| Benzothiazole | 0.83±0.02 | 75±8 | 130±8 | 110±3 |
| Quinoline | 11±1.2 | 100±14 | 118±33 | 95±20 |
| Isoquinoline | 28±0.58 | 106±6 | 109±2 | 112±1 |
| 2-Methylquinoline | 8.6±0.27 | 111±3 | 113±2 | 114±1 |
| 8-Methylquinoline | 4.3±0.4 | 119±4 | 123±0 | 124±1 |
| 6-Methylquinoline | 3.5±0.38 | 125±4 | 126±4 | 129±1 |
| 3-Methylquinoline | 3.5±0.37 | 125±4 | 129±3 | 129±1 |
| 4-Methylquinoline | 0.47±0.03 | 114±17 | 126±4 | 116±10 |
| 2,6-Dimethylquinoline | 0.51±0.06 | 125±14 | 119±7 | 131±11 |
| 2,4-Dimethylquinoline | 1.8±0.05 | 106±5 | 107±1 | 110±2 |
| Dimethyl-phthalate | 0.24±0.05 | 75±18 | 57±9 | 41±5 |
| 1-Chloro-2,4-dinitrobenzene | 9.4±0.83 | 128±13 | 130±8 | 131±3 |
| 4-Chloro-2-nitroaniline | 5.4±0.2 | 0 | 0 | 0 |
| 4-Nitroaniline | 5.2±0.5 | 106±7 | 111±4 | 118±4 |
| 2-Chloro-4-nitroaniline | 3.9±1 | 127±20 | 147±4 | 150±12 |
| Benzyl-benzoate | 11±8 | 7±1 | 8±1 | 7±1 |
| 2-Chloro-4,6-dinitroaniline | 258±18 | 107±16 | 123±6 | 127±2 |
| 2,6-Dibromo-4-nitroaniline | 4.1±0.58 | 123±8 | 132±3 | 135±2 |
| 2-Bromo-4,6-dinitroaniline | 5.6±0.93 | 97±9 | 114±16 | 117±8 |

**Table S7**: The quantified amount of target substances and the ratio of the amount after one, five, and ten laundry cycles in garment 3

| Compounds | Amount (µg/g) | 1 Cycle (%) | 5 Cycles (%) | 10 Cycles (%) |
| --- | --- | --- | --- | --- |
| Benzothiazole | 0.75±0.03 | 83±18 | 117±18 | 153±4 |
| Quinoline | 0.86±0.33 | 71±3 | 56±4 | 86±7 |
| Isoquinoline | 0.98±0.95 | 22±3 | 16±1 | 19±1 |
| Dimethyl-phthalate | 0.27±0.05 | 51±49 | 10±1 | 38±6 |
| Dimethyl-terephthalate | 0.25±0.01 | 55±2 | 81±37 | 116±14 |
| 4-Nitroaniline | 0.14±0.01 | 96±18 | 131±10 | 113±8 |
| 2-Chloro-4-nitroaniline | 31±3.3 | 90±11 | 80±10 | 75±0 |
| 2,6-Dichloro-4-nitroaniline | 0.82±0.07 | 79±12 | 64±9 | 56±1 |
| Benzyl-benzoate | 1.0±0.15 | 43±2 | 41±2 | 31±1 |
| Diisobutyl phthalate | 0.8±0.15 | 63±3 | 43±5 | 27±2 |
| 2-Chloro-4,6-dinitroaniline | 45.4±6 | 13±3 | 9±2 | 9±1 |
| 2-Bromo-4,6-Dinitroaniline | 1.4±0.23 | 0 | 0 | 0 |

**Table S8**: The quantified amount of target substances and the ratio of the amount after one, five, and ten laundry cycles in garment 6

| Compounds | Amount (µg/g) | 1 Cycle (%) | 5 Cycles (%) | 10 Cycles (%) |
| --- | --- | --- | --- | --- |
| Benzothiazole | 1.4±0.06 | 283±8 | 151±8 | 174±134 |
| Quinoline | 0.14±0.01 | 96±6 | 69±0 | 55±4 |
| Dimethyl-phthalate | 0.64±0.07 | 22±5 | 18±0 | 16±1 |
| Dimethyl-terephthalate | 0.38±0.02 | 0 | 0 | 0 |
| 2,6-Dichloro-4-nitroaniline | 1.0±0.25 | 0 | 0 | 0 |
| Benzyl-benzoate | 4.1±0.49 | 104±2 | 75±2 | 45±9 |
| Diisobutyl phthalate | 6.8±1.5 | 67±1 | 40±1 | 19±1 |

**Table S9**: The quantified amount of target substances and the ratio of the amount after one, five, and ten laundry cycles in garment 7

| Compounds | Amount (µg/g) | 1 Cycle (%) | 5 Cycles (%) | 10 Cycles (%) |
| --- | --- | --- | --- | --- |
| Benzothiazole | 1.1±0.03 | 157±3 | 150±9 | 222±64 |
| Quinoline | 0.13±0.01 | 82±11 | 72±8 | 96±19 |
| Dimethylphthalate | 0.39±0.02 | 33±2 | 26±2 | 27±5 |
| Dimethylterephthalate | 0.40±0.02 | 14±1 | 14±1 | 38±15 |
| 2,6-Dichloro-4-nitroaniline | 0.38±0.08 | 0 | 0 | 0 |
| Benzyl-benzoate | 3.3±0.28 | 52±6 | 34±3 | 23±2 |
| Diisobutyl phthalate | 2.2±0.24 | 67±6 | 38±5 | 18±2 |

**Table S10**: The quantified amount of target substances and the ratio of the amount after one, five, and ten laundry cycles in garment 11

| Compounds | Amount (µg/g) | 1 Cycle (%) | 5 Cycles (%) | 10 Cycles (%) |
| --- | --- | --- | --- | --- |
| Benzothiazole | 3.4±0.14 | 298±9 | 3363±189 | 2001±300 |
| Quinoline | 0.1±0.02 | 98±4 | 67±4 | 67±6 |
| Dimethyl-phthalate | 0.48±0.03 | 4±0 | 3±1 | 5±3 |
| Dimethyl-terephthalate | 0.27±0 | 44±5 | 42±3 | 50±11 |
| Benzyl-benzoate | 5.6±0.43 | 147±4 | 67±5 | 47±4 |
| Diisobutyl phthalate | 3.4±0.7 | 77±3 | 38±2 | 14±2 |

**Table S11**: The quantified amount of target substances and the ratio of the amount after one, five, and ten laundry cycles in garment 28

| Compounds | Amount (µg/g) | 1 Cycle (%) | 5 Cycles (%) | 10 Cycles (%) |
| --- | --- | --- | --- | --- |
| Benzothiazole | 1.4±0.05 | 125±7 | 103±11 | 163±19 |
| Quinoline | 0.24±0.02 | 76±1 | 58±18 | 75±12 |
| Dimethyl-phthalate | 0.20±0.01 | 46±8 | 10±1 | 11±1 |
| Dimethyl-terephthalate | 0.25±0.05 | 22±5 | 14±9 | 17±3 |
| Benzyl-benzoate | 1.8±0.07 | 86±14 | 37±1 | 33±1 |
| Diisobutyl phthalate | 0.86±0.04 | 102±5 | 52±1 | 34±4 |

**Table S12**: The quantified amount of target substances and the ratio of the amount after one, five, and ten laundry cycles in garment 32

| Compounds | Amount (µg/g) | 1 Cycle (%) | 5 Cycles (%) | 10 Cycles (%) |
| --- | --- | --- | --- | --- |
| Benzothiazole | 0.57±0.04 | 94±5 | 97±9 | 104±4 |
| Quinoline | 0.85±0.05 | 125±3 | 87±4 | 114±8 |
| Isoquinoline | 0.36±0.02 | 178±8 | 158±4 | 163±8 |
| 2-Methylquinoline | 0.10±0.01 | 128±4 | 123±7 | 112±3 |
| 6-Methylquinoline | 0.064±0 | 197±11 | 201±9 | 190±15 |
| Dimethyl-phthalate | 0.16±0.02 | 59±6 | 45±30 | 44±33 |
| Dimethyl-terephthalate | 0.95±0.13 | 110±25 | 331±12 | 201±36 |
| 2-Chloro-4-nitroaniline | 55±4.9 | 112±7 | 149±9 | 137±10 |
| 2,6-Dichloro-4-nitroaniline | 25±3.2 | 122±6 | 212±14 | 170±8 |
| Benzyl-benzoate | 1.9±0.07 | 38±5 | 41±4 | 25±3 |
| 2-Chloro-4,6-dinitroaniline | 124±10 | 150±12 | 276±44 | 236±4 |

**Table S13**: The quantified amount of target substances and the ratio of the amount after one, five, and ten laundry cycles in garment 45

| Compounds | Amount (µg/g) | 1 Cycle (%) | 5 Cycles (%) | 10 Cycles (%) |
| --- | --- | --- | --- | --- |
| Benzothiazole | 1.1±0.11 | 74±9 | 136±3 | 173±18 |
| Quinoline | 0.22±0.03 | 79±2 | 65±1 | 69±10 |
| Isoquinoline | 0.092±0.01 | 74±8 | 80±4 | 62±3 |
| Dimethyl-phthalate | 0.25±0.02 | 21±3 | 11±0 | 24±11 |
| Dimethyl-terephthalate | 0.29±0.01 | 31±5 | 24±1 | 28±8 |
| 2,6-Dichloro-4-nitroaniline | 0.42±0.11 | 115±19 | 101±2 | 80±12 |
| Benzyl-benzoate | 4.7±0.12 | 70±6 | 66±3 | 58±2 |
| Diisobutyl phthalate | 1.0±0.05 | 77±2 | 48±0 | 26±3 |
